# COLD-PCR enhanced melting curve analysis improves diagnostic accuracy for *KRAS *mutations in colorectal carcinoma

**DOI:** 10.1186/1472-6890-10-6

**Published:** 2010-11-26

**Authors:** Colin C Pritchard, Laura Akagi, Poluru L Reddy, Loren Joseph, Jonathan F Tait

**Affiliations:** 1 Department of Laboratory Medicine, University of Washington, Seattle, USA; 2 Department of Pathology, University of Chicago, Chicago, USA

## Abstract

**Background:**

*KRAS *mutational analysis is the standard of care prior to initiation of treatments targeting the epidermal growth factor receptor (*EGFR*) in patients with metastatic colorectal cancer. Sensitive methods are required to reliably detect *KRAS *mutations in tumor samples due to admixture with non-mutated cells. Many laboratories have implemented sensitive tests for *KRAS *mutations, but the methods often require expensive instrumentation and reagents, parallel reactions, multiple steps, or opening PCR tubes.

**Methods:**

We developed a highly sensitive, single-reaction, closed-tube strategy to detect all clinically significant mutations in *KRAS *codons 12 and 13 using the Roche LightCycler^® ^instrument. The assay detects mutations via PCR-melting curve analysis with a Cy5.5-labeled sensor probe that straddles codons 12 and 13. Incorporating a fast COLD-PCR cycling program with a critical denaturation temperature (*T_c_*) of 81°C increased the sensitivity of the assay >10-fold for the majority of *KRAS *mutations.

**Results:**

We compared the COLD-PCR enhanced melting curve method to melting curve analysis without COLD-PCR and to traditional Sanger sequencing. In a cohort of 61 formalin-fixed paraffin-embedded colorectal cancer specimens, 29/61 were classified as mutant and 28/61 as wild type across all methods. Importantly, 4/61 (6%) were re-classified from wild type to mutant by the more sensitive COLD-PCR melting curve method. These 4 samples were confirmed to harbor clinically-significant *KRAS *mutations by COLD-PCR DNA sequencing. Five independent mixing studies using mutation-discordant pairs of cell lines and patient specimens demonstrated that the COLD-PCR enhanced melting curve assay could consistently detect down to 1% mutant DNA in a wild type background.

**Conclusions:**

We have developed and validated an inexpensive, rapid, and highly sensitive clinical assay for *KRAS *mutations that is the first report of COLD-PCR combined with probe-based melting curve analysis. This assay significantly improved diagnostic accuracy compared to traditional PCR and direct sequencing.

## Background

KRAS (Kirsten rat sarcoma virus homolog 2) is a membrane-anchored G-protein that acts downstream of the epidermal growth factor receptor (EGFR) to activate pro-growth and anti-apoptotic pathways, including the MAP kinase and PI3 kinase pathways [1]. Mutations in codons 12 and 13 of the *KRAS *gene confer resistance to drugs targeted at EGFR by impairing GTPase activity, which results in constitutive EGFR-independent signaling. *KRAS *is one of the most frequently mutated oncogenes in human cancer, with significant mutation rates in common epithelial malignancies such as colon cancer (~40%), lung cancer (~20%), gastric cancer (~10%), and pancreatic cancer (~65%) (COSMIC database; http://www.sanger.ac.uk/genetics/CGP/cosmic/) [2,3].

The role of *KRAS *mutation status in clinical decision making is best defined for colon cancer. Several large randomized-controlled trials demonstrated no benefit from expensive anti-EGFR drugs such as cetuximab (Erbitux™) in patients with *KRAS*-mutant colon cancer [4,5]. The National Comprehensive Cancer Network (NCCN) now recommends *KRAS *testing prior to initiation of anti-EGFR therapy in colon cancer patients [6]. Accumulating evidence also suggests a role for *KRAS *testing to guide therapy in patients with non-small cell lung cancer [7,8].

Greater than 95% of *KRAS *mutations occur in codon 12 or codon 13. Within these codons G12 D (GGT to GAT), G12V (GGT to GTT), and G13 D (GGC to GAC) comprise ~80% of the mutations [2,4]. Less frequent mutations include G12 S (GGT to AGT), G12C (GGT to TGT), G12R (GGT to CGT), and G12A (GGT to GCT). Silent mutations are exceedingly rare http://www.sanger.ac.uk/genetics/CGP/cosmic/.

CO-amplification at Lower Denaturation temperature (COLD)-PCR is a recently described method to selectively amplify mutant alleles in a wild type background that does not require any additional instrumentation or reagents to implement [9]. Two forms of COLD-PCR are described: *fast *COLD-PCR and *full *COLD-PCR (reviewed in [10]). Fast COLD-PCR enriches G:C to A:T mutations that slightly, but predictably lower the melting temperature (*T_m_*) of the PCR amplicon, by using a critical denaturation temperature (*T_c_*) that favors PCR amplification of the mutant allele. Full COLD-PCR theoretically enhances detection of any type of mutation via conditions which promote annealing of WT:mutant pairs and selective denaturation of these heteroduplexes at an empirically determined *T_c_*. As the name implies, fast COLD-PCR has the advantage of being more rapid than full COLD-PCR (1-2 hours of instrument time compared to 5-8 hours) and is also easier to troubleshoot and implement in our experience. Fast COLD-PCR is ideal for *KRAS *codon 12 and 13 mutational analysis because >90% of the mutations are G:C to A:T changes (G12 D, G12V, G12 S, G12C, G13D), and the remaining common mutations are *T_m _*-neutral G:C to G:C changes (G12R and G12A).

Here, we report a novel strategy for mutation detection that combines fast COLD-PCR with two-probe melting curve analysis using the LightCycler^® ^instrument. By incorporating COLD-PCR we achieve >10-fold enhancement for the majority of *KRAS *mutations with a sensitivity of ~1% mutant allele in a wild type background.

## Methods

### Patient tumor samples and DNA extraction

All clinical specimens consisted of DNA prepared from formalin-fixed paraffin-embedded (FFPE) tissue from patients with sporadic colorectal carcinoma. De-identified residual clinical specimens were obtained in accordance with the declaration of Helsinki and ethics guidelines of the local institutional review board. We obtained a total of 61 de-identified specimens: 10 DNA specimens from the University of Texas MD Anderson Cancer Center, 4 specimens of unstained FFPE tissue sections with corresponding hematoxylin-eosin (HE) stained slides from the University of Chicago, and 47 DNA specimens prepared from FFPE tissue from colorectal cancer specimens at the University of Washington. HE-stained slides were used as a guide to manually dissect areas of tumor tissue from unstained slide sections. Genomic DNA was prepared with the Gentra Puregene DNA Isolation Kit (Qiagen, Cat. No. 158489).

### KRAS mutational detection by Regular- and COLD-PCR enhanced melting curve analysis

**Figure 1:**
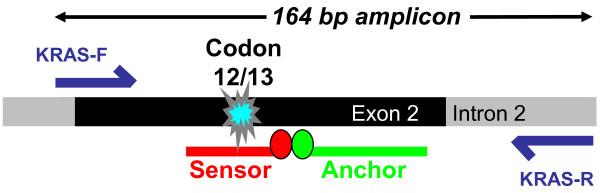
**Melting Curve Assay Design**. PCR primers are designed within introns of the functional *KRAS *(Ki-Ras2) gene on chromosome 12 to avoid amplification of a *KRAS *(Ki-Ras1) pseudogene on chromosome 6. A Cy5.5-labeled wild type sensor probe sits over the mutation hotspot at codons 12 and 13. When a *KRAS *mutation is present, the sensor probe melts off at a lower *T_m_*.

Our basic melting-curve strategy to identify *KRAS *mutations is a modification of a procedure described by Nikiforova and colleagues [11]. The assay is a PCR fluorescence resonance energy transfer (FRET) hybridization probe assay performed with a LightCycler^® ^carousel instrument (Roche Molecular Biochemicals, Manheim Germany). This format requires a pair of primers and two fluorescently labeled oligonucleotide probes and relies on FRET between two probes that hybridize adjacent to one another (Figure 1). For the detection of KRAS codon 12/13 mutations, the Cy5.5-labeled probe spans this region, and the products are distinguished based on their distinct *T_m_*, which reflects the thermodynamic stability of the perfectly complementary and mismatched probe-target duplexes. The wild-type *T_m _*is 69.5 ± 0.5°C, and the mutant *T_m _*is 62.8 ± 1°C. The assay cannot reliably determine the exact base change based on the *T_m_*. The primers and probes are given in Table 1. Each 20 μl PCR reaction contained 2 μl 10× LightCycler^® ^FastStart DNAHyb Master mix (Roche Cat No. 03 002248 001), 0.5 units of LightCycler^® ^uracil N-glycosylase enzyme (UNG) (Roche Cat No. 03539806001), 2 mM final MgCl_2 _concentration, 30 pmol KRAS-F (1.5 μM final concentration), 10 pmol KRAS-R (0.5 μM final concentration), 4 pmol anchor probe (0.2 μM final concentration), and 4 pmol sensor probe (0.2 μM final concentration). Template DNA was added at 10-200 ng per reaction. PCR reactions were set up in glass LightCycler^® ^Capillaries (Roche Cat. No. 1 909 339) and run on both LightCycler^® ^1.5 and LightCycler^® ^2.0 carousel instruments. For the regular PCR melting curve assay (not COLD-PCR enhanced) the cycling program steps were as follows: 1) UNG step to remove carry-over amplicon, 40°C for 10 min, 2) activation of antibody-inhibited Taq polymerase, 94°C for 10 min, 3) cycling 94°C 1 s, 52°C 20 s, 72°C 10 s for 45 total cycles, 4) melting curve with continuous temperature ramping at 0.15°C/s from 45°C to 80°C, and 5) Cooling to 40°C. The COLD-PCR enhanced cycling program is shown in Table 2. An initial 10 cycles of regular PCR are performed to build up product, followed by 40 cycles of COLD-PCR with a critical denaturation temperature (*T_c_*) of 81°C which favors amplification of mutant alleles. We determined the *T_c _*empirically by progressively lowering the denaturation temperature from 84°C → 82°C → 81°C → 80°C → 79°C. Melting curves were produced in both mutant and wild type samples down to *T_c _*= 80°C. We chose the *T_c _*at 81°C because the melting curves were difficult to interpret at *T_c _*= 80°C due to reduced PCR efficiency and increased signal-to-noise ratio (Additional file 1 Figure S1).

**Table 1:** Primer and Probe Sequences (adapted from [11])

Primer/Probe	Sequence	Position	Chr. 12
KRAS-F	5'-AAG GCC TGC TGA AAA TGA CTG-3'	Intron 1/Exon 2 boundary	25398312-25398332
KRAS-R	5'-GGT CCT GCA CCA GTA ATA TGC A-3'	Intron 2	25398168-25398189
Anchor Probe	5'-CGT CCA CAA AAT GAT TCT GAA TTA GCT GTA TCG TCA AGG CAC T-fluorescein-3'	Exon 2	25398228-25398270
Sensor Probe	5'-Cy5.5-TGC CTA CGC CAC CAG CTC CAA-phosphate-3'	Exon 2 spanning codons 12/13	25398273-25398293

Chr.12: genomic numbering on chromosome 12, human genome build 19 (hg19, February 2009 build).

**Table 2:** Fast COLD-PCR Cycling Program

Step	Temp	Time	Analysis Mode	Acquisition Mode
UNG	40°C	10 m	None	None
				
Denaturation	94°C	10 m	None	None
				
Regular-PCR (10 cycles)	94°C	1 s	None	None
	52°C	20 s	None	None
	72°C	10 s	None	None
				
COLD-PCR (40 cycles)	**81 **°C **(*T_c_*)**	1 s	None	None
*Ramp 2.0°C/s*	52°C	20 s	Quant	Single
	72°C	10 s	None	None
				
Melting curve	94°C	5 s	None	None
	45°C	30 s	None	None
	80°C	Ramp 0.15°C/s	Melting	Continuous
				
Cooling	40°C	10 s	None	None

UNG: Uracil-N-Glycosylase; Temp: Temperature.

### Regular- and COLD-PCR enhanced Sanger DNA sequencing

We used KRAS-F and KRAS-R primers (Table 1) for both PCR amplification and DNA sequencing. For DNA sequencing, regular- or COLD-PCR reactions were performed as described above except that the anchor and sensor probes were not added to the reactions and thermocycling was performed on a Bio-Rad C1000 instrument. PCR products were purified using DNA Clean & Concentrator™-5 (Zymo Research) and eluted in a 30 μl volume of elution buffer (Cat No D4003, D4004, D4013 & D4014). Each 10 μl sequencing reaction included 2 μl Big Dye Terminator v3.1 (Applied Biosystems, ABI) 2 μl 5× sequencing buffer (ABI), 4 pmol of sequencing primer (either KRAS-F or KRAS-R), and 200-300 ng purified PCR product. Sequencing reactions were cleaned up using AutoSeq G-50 Sephadex columns (Amersham Biosciences Cat. No. 27-5340), run on an ABI 3130 instrument, and analyzed with Mutation Surveyor software (Softgenetics).

We sequenced a total of 30 out of 61 samples including all 4 discrepant samples that were re-classified as *KRAS*-mutant by the COLD-PCR melting curve analysis (Additional file 2 Table S1). Two of the four discrepant samples had very low mutant peaks by COLD-PCR sequencing using the same thermocycling conditions (*T_c_*= 81°C, Table 2) that produced easily discernable mutant peaks in the COLD-PCR melting curve assay, presumably because the melting curve analysis is inherently more sensitive than sequencing. For this reason, we lowered the *T_c _*to 79.4°C to further enhance amplification of minority mutant alleles and confirm the *KRAS *mutations we detected in the 4 discrepant samples. We selected the *T_c _*of 79.4°C after we tried *T_c _*ranging from 79-81°C at 0.2°C increments and determined that the *T_c _*of 79.4°C was the lowest denaturation temperature in which PCR product from a wild type sample was still visible an agarose gel. We repeated COLD-PCR sequencing with *T_c _*= 79.4°C a total of 3 times for each of the 4 discrepant samples, obtaining concordant *KRAS*-mutant results and appropriately negative *KRAS*-wild type controls on each run.

### Confirmation of Discrepant Specimens With An Alternative Assay

In addition to COLD-PCR sequencing, we confirmed the 4 discrepant specimens using the Lightmix *KRAS *mutation detection kit following the manufacturer's instructions (TIB Molbiol, Cat#04-0416-08). This assay uses a locked nucleic acid oligomer competitor and has a stated sensitivity of at least 1% for mutant allele in a wild type background (Additional file 1 Figure S2).

### Mixing/Sensitivity Studies

We performed two types of mixing studies to estimate sensitivity: (1) *KRAS*-mutant colorectal cancer cell lines mixed with *KRAS*-WT peripheral blood leukocyte DNA, and (2) *KRAS*-mutant FFPE DNA mixed with *KRAS*-WT FFPE DNA. We used a total of three *KRAS*-mutant colorectal cancer cell lines, SW480 (homozygous G12V; GGT to GTT), HCT116 (heterozygous G13D; GGC to GAC) and LS174T (heterozygous G12D; GGT to GAT), and two *KRAS*-mutant FFPE colorectal cancer samples (one G12 D and one G13D). The undiluted G12 D and G13 D FFPE samples both had ~ 25% *KRAS *mutant allele based on a visual estimate of tumor nuclear content and on the mutant:WT peak height ratios in regular PCR melting curve analysis (data not shown). To control for potential differences in ploidy between samples and variation in DNA preparations that may confound the interpretation of mixing studies, we first adjusted each DNA preparation so that the cycle thresholds (Ct) of all mutant and WT samples were within 0.5 of each other (PCR-efficiency matched). Mutant cell lines or FFPE DNA samples were mixed in the following ratios (mutant%/WT%): 50/50, 20/80, 10/90, 5/95, 2/98, 1/99. We performed mixing studies in duplicate on a total of 5 unique mutant:WT pairs.

As an alternative measure of sensitivity, we tested 20% (G13C) and 1% (G12C) *KRAS*-mutant control DNA provided with the Lightmix *KRAS *mutation detection kit (TIB Molbiol, cat # 04-0416-08, Lot#4160902).

## Results

### Establishing and Validating COLD-PCR Conditions

**Figure 2:**
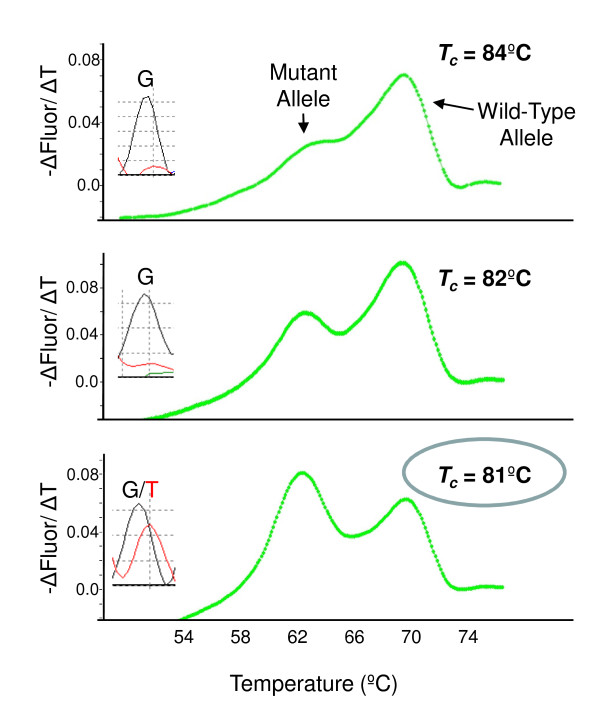
**Optimization of COLD-PCR Conditions**. A G12C (GGT-TGT) *KRAS*-mutant colorectal cancer sample with approximately 10% mutant allele was tested at progressively lower critical denaturation temperatures (*T_c_*). As the *T_c _*was progressively lowered, the height of the mutant melting peak at 62.5°C increased relative to the height of the wild-type melting peak at 70°C. DNA sequencing performed on COLD-PCR products corroborated an increase in the peak height of the mutant *T *base (c.34 G > T) at the first position of codon 12 (compare top and bottom panel insets).

To establish the critical denaturation temperature (*T_c_*) for COLD-PCR melting curve analysis we selected a G12C *KRAS*-mutant FFPE colorectal cancer specimen with ~10% mutant allele based on a visual estimate of tumor nuclei and the mutant/WT melting peak height ratio using regular PCR. The mutant/WT peak height ratio increased by an average of 6.3-fold in this "low-positive" patient sample as the *T_c _*was lowered from 84°C → 82°C → 81°C (Figure 2). COLD-PCR DNA sequencing confirmed a proportional increase in the mutant *T *base at the first position of codon 12 (G12C = GGT→TGT) as the *T_c _*was lowered (Figure 2 insets). We found that using a *T_c _*lower than 81°C further enhanced the relative proportion of mutant allele, but reduced PCR efficiency to a degree that made it more difficult to interpret melting peaks (Additional file 1 Figure S1).

We next validated the COLD-PCR melting curve assay using FFPE colorectal cancer specimens with distinct characterized *KRAS *mutations. COLD-PCR enhanced detection of G:C to A:T mutations (G12 D, G13 D, G12C, G12V) between 9.8 and 14.0 fold compared to regular PCR (Summarized in Table 3). Importantly, the COLD-PCR reaction conditions we established did not diminish detection of *T_m _*-neutral G:C to G:C mutations (G12R and G13R). Surprisingly, the fast COLD-PCR reaction conditions actually enhanced the detection of a G13R (GGC to CGC) mutation, which we did not expect to enhance based on a *T_m _*-neutral G > C change. We hypothesize that this enrichment is due to local sequence effects or to a small amount of WT:mutant heteroduplex formation during the primer annealing step, which favors amplification of mutant alleles by the same mechanism as full COLD-PCR (see Background for discussion of fast vs. full COLD-PCR) [10].

**Table 3:** COLD-PCR Enhancement According to Specific *KRAS *Mutation

Mutation	Base Change	Frequency in Colorectal Cancer	Predicted To Enhance With Fast COLD-PCR?	Fold-Enhancement With COLD-PCR
G12D	GGT to GAT	35%	Yes	11.5
G12V	GGT to GTT	22%	Yes	11.5
G13D	GGC to GAC	19%	Yes	9.8
G12C	GGT to TGT	9%	Yes	14.0
G12S	GGT to AGT	7%	Yes	ND
G12A	GGT to GCT	7%	No	ND
G12R	GGT to CGT	1%	No	1.1
G13R	GGC to CGC	< 1%	No	2.5
Other	Multiple	< 1%	Yes/No	ND

ND = no data; Frequency Is Based on *KRAS *Mutations Colorectal Cancer Reported in the COSMIC Database accessed July 2010 http://www.sanger.ac.uk/perl/genetics/CGP.

### Sensitivity of the COLD-PCR Enhanced Melting Curve Assay

**Figure 3:**
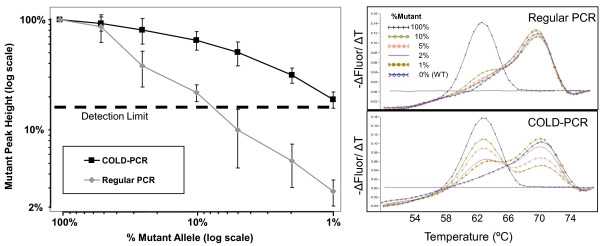
**Sensitivity of the Assay**. Five independent mixing studies were performed in duplicate using *KRAS*-mutant cell lines (SW480, LS174T, HCT116) paired with wild-type peripheral blood leukocyte DNA and *KRAS*-mutant formalin-fixed paraffin-embedded (FFPE) tissue samples paired with *KRAS*-WT FFPE samples as described in the methods section. For each mixing study the mutant melting peak height was measured as a percentage of the maximum mutant peak height observed in the undiluted *KRAS*-mutant sample specific to the particular Mutant:WT pairing. The left panel shows the average and SD (error bars) of the %mutant peak height across the 5 mixing studies using regular- (gray triangles) or COLD-PCR (black squares) conditions. Mutant peak heights were consistently above the limit of detection down to 1% mutant allele using COLD-PCR compared to 10% mutant allele with regular PCR. The right panel shows an example of one of the mixing studies (SW480 cell line; homozygous G12V) used to generate the sensitivity graph in the left panel. Mutant melting peaks are not clearly discernible for 1%, 2%, and 5% *KRAS*-mutant samples using regular PCR (top right panel), but are readily detected using COLD-PCR (bottom right panel).

To determine the sensitivity of the COLD-PCR melting curve assay to detect low quantities of mutant allele in WT background we adopted two complementary strategies: (1) we mixed *KRAS*-mutant cell line DNA with *KRAS*-WT peripheral blood leukocyte DNA, and (2) *KRAS*-mutant FFPE tumor DNA with *KRAS*-WT FFPE tumor DNA at mutant:WT ratios of 50/50, 20/80, 10/90, 5/95, 2/98, and 1/99. Prior to mixing we ensured the PCR-efficiency of each sample in a pair was matched within 0.5 Ct because variations in DNA quality, ploidy, and sample preparation may favor the amplification of one sample over the other and confound the estimation of sensitivity [12]. In a total of 5 unique sample pairings which included G12 D, G13 D, and G12V-mutant DNA, the assay could readily detect KRAS mutations down to 1% mutant allele in each case with COLD-PCR enhancement, but only down to 10% mutant allele with regular PCR (Figure 3).

As a second measure of sensitivity, we ran commercial 20% (G13C) and 1% (G12C) *KRAS*-mutant control DNA (TIB Molbiol, Lot No. 4160902). The COLD-PCR enhanced melting curve assay correctly classified both controls as *KRAS*-mutant with a mutant:WT peak height ratio of 3.5 for the 20% G13C control, and 0.3 for the 1% G12C control (Additional file 1 Figure S3).

**Figure 4:**
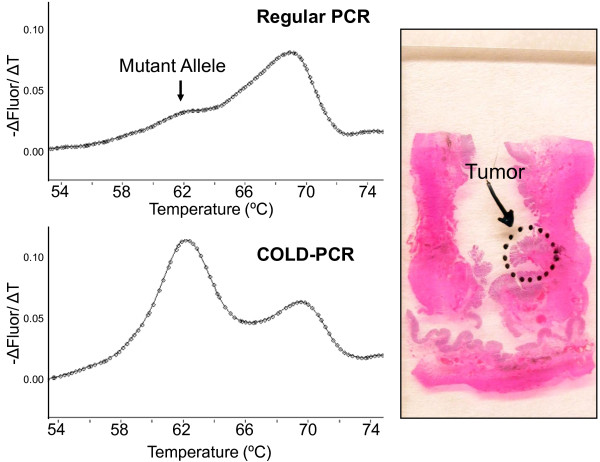
***KRAS *Mutation Detection Without Dissection**. DNA was prepared from a G12 D *KRAS*-mutant colorectal cancer resection specimen with ~3% tumor cells by scraping the tissue from the entire slide without tumor dissection. The *KRAS *result was equivocal using regular-PCR (upper left panel), but a mutation was clearly detected using COLD-PCR (lower left panel). The corresponding HE-stained slide and region of tumor are depicted in the right panel.

We next assessed if the COLD-PCR enhanced melting curve assay was sensitive enough to eliminate the need for tumor dissection in a *KRAS*-mutant sample with a low percentage (~3%) of tumor cells. We confirmed that both regular-PCR melting curve analysis and Sanger sequencing detected a *KRAS *mutation (G12D) when DNA was prepared with morphology-guided dissection to enrich for tumor cells in our standard fashion. We then tested a DNA sample prepared from the same tumor specimen without dissection (scraping the whole slide). The COLD-PCR enhanced melting curve assay readily detected the *KRAS *mutation when the whole slide was scraped, whereas the result with regular PCR melting curve analysis was equivocal (Figure 4).

### COLD-PCR Enhanced Melting Curve Analysis Detects Mutations That Are Missed By Regular PCR and Direct Sanger Sequencing

**Figure 5:**
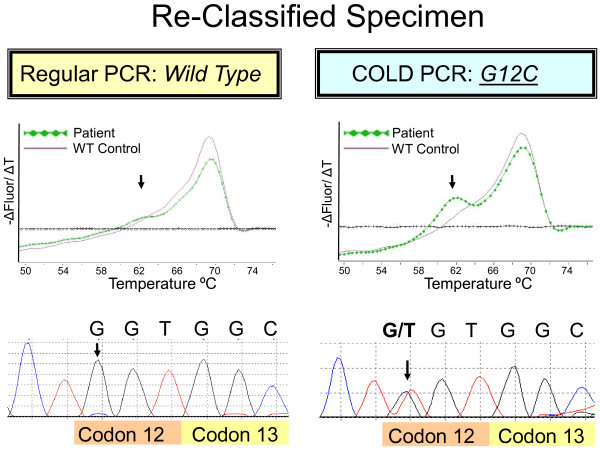
**Re-classified specimen**. Shown are results from one of the 4 specimens that was re-classified from *KRAS*-WT to *KRAS*-mutant by COLD-PCR (sample #3 in Additional file 3). With regular PCR no definite mutant allele is detected by the melting curve assay, or by direct sequencing (left panel). Using COLD-PCR a clear mutant peak is visible in the melting curve assay and confirmed as a G12C mutation by sequencing (c.34 G > C). Arrows point to the position of the mutant melting peak and mutant base pair position. Note that a *T_c _*of 79.4°C was used for COLD-PCR sequencing compared to a *T_c _*of 81°C for the COLD-PCR melting curve assay (see Methods), which is the likely explanation for the higher proportion of mutant allele observed by COLD-PCR sequencing (bottom right).

We compared the COLD-PCR melting curve assay to regular PCR melting analysis in a cohort of 61 FFPE colorectal cancer specimens which included 14 blinded samples that had *KRAS *testing done previously at outside institutions. *KRAS *mutations were detected in 29/61(48%) samples by both regular- and COLD PCR melting curve analysis and, importantly, in an additional 4 samples (33/61 total; 54%) by COLD-PCR enhanced melting curve analysis only (Table 4; Additional file 2 Table S1). Regular Sanger sequencing did not detect mutations in the 4 samples that were re-classified from *KRAS*-WT to *KRAS*-mutant by COLD-PCR, presumably because the mutant allele % was below the limit of detection (sensitivity ~20 percent for standard sequencing). However, COLD-PCR enhanced DNA sequencing confirmed that the 4 re-classified samples each harbored a clinically significant *KRAS *mutation (Additional file 3; Figure 5; see methods section for a detailed discussion of COLD-PCR sequencing). *KRAS *mutations in the 4 re-classified samples were also confirmed using the TIB Molbiol Lightmix assay, which has a stated sensitivity of at least 1% (Additional file 1 Figure S2). Sequencing of an additional 26 specimens that were concordant by regular- and COLD-PCR confirmed the *KRAS *melting curve analysis result in all cases (Additional file 2 Table S1).

**Table 4:** Comparison of Regular- and COLD-PCR in 61 FFPE Samples

		**COLD-PCR**			
		
		**Pos**	**Lo-Pos**	**WT**	**Total**
		
**Regular PCR**	Pos	19	0	0	19
		
	Lo-Pos	10	0	0	10
		
	WT	4	0	28	32
		
	Total:	33	0	28	61

FFPE: Formalin-fixed paraffin-embedded Pos: Positive for *KRAS *mutation; Lo-Pos: Positive for *KRAS *mutation near the limit of detection; WT: Wild Type

Among the 14 specimens from outside institutions, *KRAS *results were concordant using the less sensitive regular PCR melting curve analysis and Sanger sequencing (7/14 *KRAS*-mutant; 7/14 *KRAS*-WT). However, we re-classified 1/14 of these specimens as *KRAS*-mutant by both COLD-PCR melting curve analysis and COLD-PCR sequencing. The method used by the outside laboratory to identify *KRAS *mutations was regular sequencing, which explains why the *KRAS *mutation in that specimen was not originally detected.

## Discussion

Cancer tissue presents challenges for *KRAS *mutation analysis because each specimen is a unique mixture of tumor and non-tumor cells. A typical formalin-fixed paraffin-embedded colorectal cancer specimen might have between 2-50% tumor cells, which corresponds to 1-25% mutant allele when the tumor is diploid and a heterozygous mutation is present. To enrich the proportion of tumor cells most laboratories (including ours) manually dissect areas of tumor tissue from unstained sections guided by an HE-stained slide. This approach improves mutation detection, but tumors with a diffuse growth pattern or dense inflammatory infiltrate may still have a significant proportion of "contaminating" non-tumor DNA after tumor dissection. For this reason, methods such as COLD-PCR and peptide nucleic acid (PNA) clamping have been developed to increase the sensitivity of mutation analysis by selectively amplifying mutant alleles in a wild type background [9,10,13].

Here, we demonstrate that COLD-PCR combined with probe-based melting curve analysis can reproducibly detect down to 1% *KRAS*-mutant allele in a wild type background. This is comparable to the sensitivity reported with PNA clamping methods applied to melting curve analysis [14,15]. We show that our COLD-PCR method can readily detect the 20% and 1% *KRAS*-mutant controls used in the nucleic acid clamping-based TIB Molbiol commercial kit for the LightCycler^® ^instrument, with substantially less expensive reagents (~$5 compared to ~$100 per reaction). Limitations of our study include the relatively small sample size (n = 61), and lack of validation of the full range of rare *KRAS *mutations. A larger scale study is required to determine the assay's performance on the full complement of rare *KRAS *mutations.

A recent editorial on COLD-PCR technology suggested that "The utility of COLD-PCR will be greatly enhanced if this approach can be demonstrated to completely eliminate microdissection steps or at least to substantially reduce the tumor percentage required....[16]." We show that the COLD-PCR melting curve assay is sensitive enough to readily detect a *KRAS *mutation without tumor dissection in a sample with only ~3% tumor cells. Despite this, we believe it is prudent to continue dissection for tumor enrichment because additional mutational testing may be required on the same DNA preparation, and these add-on tests may have lower sensitivity.

Among a cohort of 61 sporadic colorectal cancer specimens we found that 4/33 (12.1%) specimens harboring *KRAS *mutations were incorrectly classified as *KRAS*-WT using regular (conventional) PCR melting curve analysis and Sanger sequencing, despite dissection of samples for tumor enrichment. An additional 10/33 *KRAS*-mutant specimens were close to the limit of detection using standard melting curve analysis (Table 4 "Lo-Pos"). These findings are supported by recent reports of false negative or equivocal *KRAS *results when less sensitive methods are used [13,17,18]. One study identified 10/57 (17.5%) additional *KRAS*-mutant samples when COLD-PCR was incorporated into high resolution melting curve analysis [17]. Kobunai and colleagues detected *KRAS *mutations in an additional 25 out of 224 colorectal tumors when a highly sensitive PNA-clamping method was compared to direct sequencing [13]. A third group reported equivocal *KRAS *results in 10% (18/180) of FFPE tumor specimens using a melting curve approach that was similar to our assay without COLD-PCR enhancement [18].

Misclassification of *KRAS *mutation status has important clinical consequences because *KRAS *testing of tumor tissue is often used as the sole determinant for selection of anti-EGFR therapy [19,20]. Taken together, our data and the results of other recent studies suggest that more than 10% of patients with *KRAS *codon 12/13 mutations are incorrectly classified as wild type when less sensitive methods are used. This has implications for patient care and for the interpretation of results from clinical trials that assessed *KRAS *mutations by direct sequencing, such as the 2008 landmark New England Journal of Medicine study by Karapetis and colleagues [4].

Previous studies have reported COLD-PCR applied to *KRAS *mutational analysis [9,17,21] and COLD-PCR combined with high-resolution melting curve analysis [17,22], but to our knowledge this is the first report of COLD-PCR combined with fluorescent probe-based melting analysis. Mancini and colleagues applied COLD-PCR to *KRAS *mutation detection using the Corbett Research RotorGene 6000 high resolution melting instrument [17]. The method described by Zuo et. al. 2009 utilized a ~7 hour full COLD-PCR protocol followed by a second reaction in a pyrosequencing instrument and achieved 1.1 to 4.7-fold enhancement *KRAS*-mutant signal compared to regular PCR [21]. Milbury and colleagues used single-step and nested PCR conditions followed by high resolution melting curve in a separate instrument [22]. There are important advantages to the novel strategy we describe here which make it appealing for the clinical setting compared to earlier reports. Most importantly, our method is performed as a single ~1 hour reaction and is entirely closed-tube, which cuts down substantially on labor and instrument time, while minimizing the risk of sample handling errors and contamination. Additionally, compared to high resolution melting curve analysis, our probe-based method offers theoretical increased specificity afforded by 2 primers and 2 probes (compared to 2 primers only), and better suitability for laboratories that already own or have experience with the Roche LightCycler^® ^instrument.

## Conclusion

We report a rapid, single-reaction, closed-tube clinical assay for *KRAS *mutations that combines COLD-PCR with melting curve analysis using the LightCycler^® ^instrument. The assay has a sensitivity of at least 1%, which is comparable to or better than many commercial kits and previous studies, although methods with superior sensitivity have been reported [22-24]. In a cohort of 61 colorectal cancer samples, the assay improved diagnostic accuracy by correctly identifying 4 *KRAS*-mutant samples that were not detected by regular melting curve analysis or direct sequencing. Incorporation of COLD-PCR with probe-based melting curve analysis was straight-forward to optimize and implement, and is therefore an appealing strategy for assays designed to detect low-level minority alleles.

## List of Abbreviations

**KRAS**: Kirsten rat sarcoma virus homolog 2; **EGFR**: Epidermal growth factor receptor; **COLD-PCR**: Co-amplification at lower denaturation temperature polymerase chain reaction; **FFPE**: Formalin-fixed paraffin-embedded; **WT**: Wild type; ***T_m_***: Melting temperature; ***T_c_***: Critical denaturation temperature; **Ct**: Cycle Threshold; **PNA**: Peptide nucleic acid

## Competing interests

The authors declare that they have no competing interests.

## Authors' contributions

CP developed the assay, designed the study, helped perform the experiments, analyzed the data, and drafted the manuscript. LA helped perform the experiments. PR and LJ helped design the study, provided FFPE specimens, commercial controls, scientific guidance, and reviewed the manuscript. JT helped design the study, analyze the data, review the manuscript, and also provided resources and scientific guidance. All authors have read and approved the final manuscript.

## Pre-publication history

The pre-publication history for this paper can be accessed here:

http://www.biomedcentral.com/1472-6890/10/6/prepub

## Supplementary Material

Additional file 1 **Supplemental Figures S1-S3**. Supplemental figures S1, S2, and S3, with figure legends. Click here for file

Additional file 2 **Table S1**. Supplemental Table 1: Complete list of mutations detected by regular- or COLD-PCR. Click here for file

Additional file 3 **Sequencing results on the re-classified specimens**. Regular- and COLD-PCR sequencing data for the 4 re-classified (discrepant) specimens. Click here for file
