# Supplementary material for: COLD-PCR enhanced melting curve analysis improves diagnostic accuracy for KRAS mutations in colorectal carcinoma
Source: BMC Clin Pathol. 2010 Nov 26;10:6. doi: 10.1186/1472-6890-10-6 (PMC3001699; doi:10.1186/1472-6890-10-6)
Supplement: Additional file 1 — Supplemental Figures S1-S3. Supplemental figures S1, S2, and S3, with figure legends. [file 1472-6890-10-6-S1.PDF]

Supplemental Figure 1

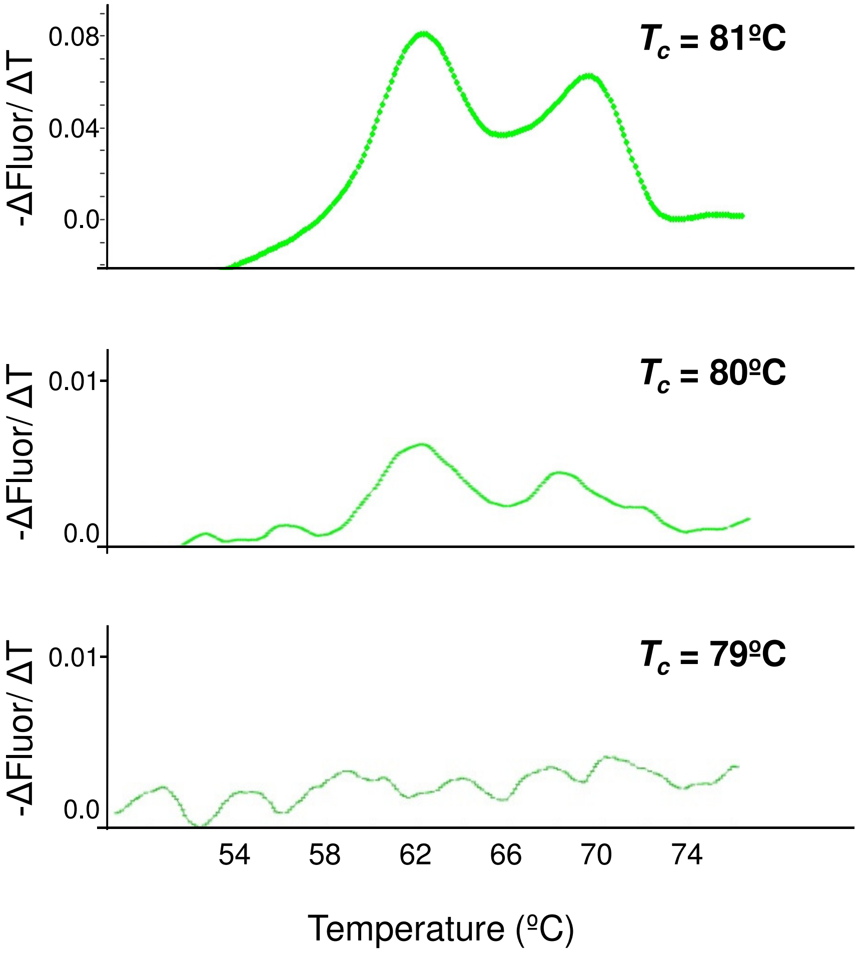

Figure S1: Performance of the melt curve assay at T_c less than 81ºC. COLD-PCR melt curve assay at optimal T_c = 81ºC, and at T_c= 80ºC and T_c= 79ºC . The efficiency of PCR drops off sharply at T_c lower than 81ºC. The specimen is a G12C (GGT-TGT) *KRAS*-mutant colorectal cancer sample with approximately 10% mutant allele.

Supplemental Figure 2

Sample #1 G12D (peak at 61.0 ºC)

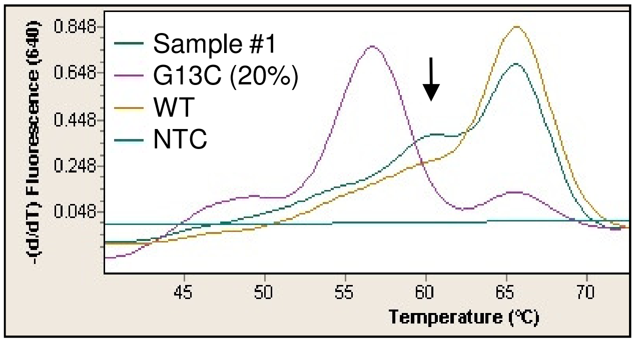

Sample #2 G13D (peak at 57.6 ºC)

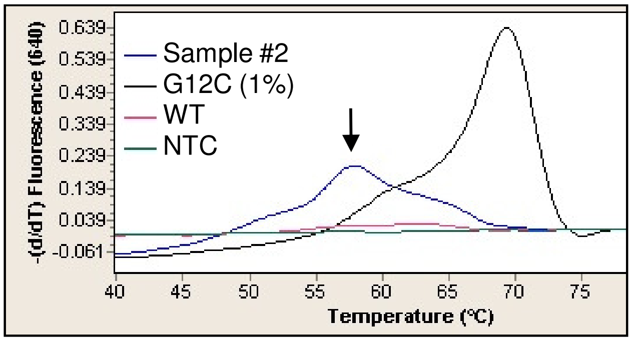

Sample #3 G12C (peak at 68.3 ºC)

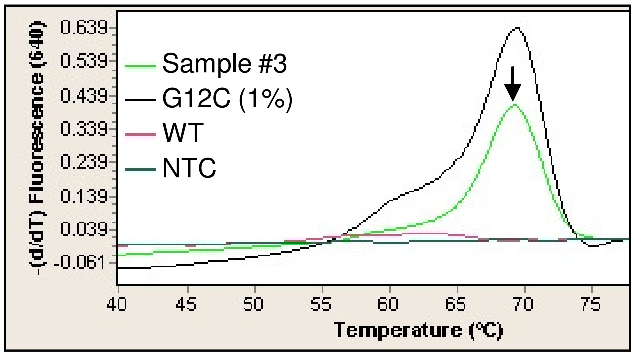

Sample #4 G13R (peak at 56 ºC)

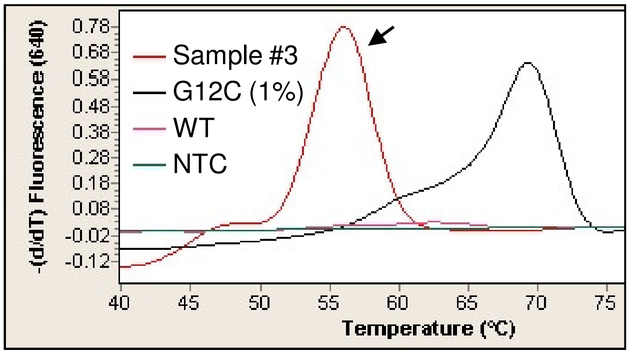

Figure S2: Re-classified (discrepant) specimens are confirmed to harbor *KRAS* mutations using LNA clamp assay. The 4 re-classified (discrepant) samples were tested with the TIB Molbiol LightMix kit, which is a melt curve assay that uses a locked nucleic acid (LNA) clamp to suppress wild type allele and enhance detection of minority mutant alleles. In this assay, the temperature of the melting peak can be used to identify the specific *KRAS* mutation. Arrows point to the melting peak corresponding to the *KRAS*-mutant allele (G12D= 61.0 ºC, G13D= 57.6 ºC, G12C= 68.3 ºC, G13R 56 ºC; WT allele is at 65 ºC). Each of the 4 re-classified specimens was confirmed to have the same *KRAS* mutation identified by COLD-PCR sequencing (see supplemental data file for sequence traces). Note that when high concentrations of LNA clamp are used, the wild type allele is completely suppressed, which is why the wild type (WT) control peak is not seen except in the top left panel, where a lower concentration of LNA clamp was used.

Supplemental Figure 3

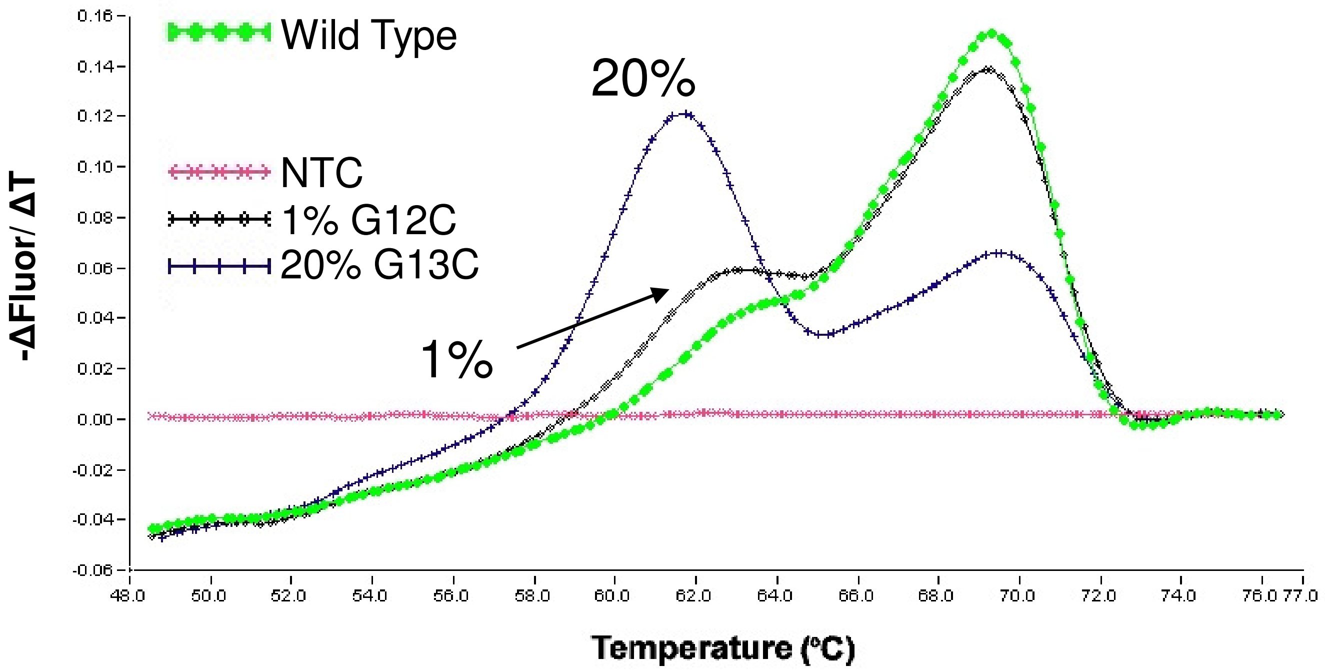

Figure S3: Performance of the assay using external controls with low quantities of KRAS mutant allele. TIB Molbiol 20% (G13C) and 1% (G12C) commercial controls were correctly classified with the COLD-PCR melting curve assay.
