# Supplementary material for: COLD-PCR enhanced melting curve analysis improves diagnostic accuracy for KRAS mutations in colorectal carcinoma
Source: BMC Clin Pathol. 2010 Nov 26;10:6. doi: 10.1186/1472-6890-10-6 (PMC3001699; doi:10.1186/1472-6890-10-6)
Supplement: Additional file 2 — Table S1. Supplemental Table 1: Complete list of mutations detected by regular- or COLD-PCR. [file 1472-6890-10-6-S2.PDF]

Supplemental Table S1: Mutation Detection in FFPE Samples

	Melting Curve		Sequencing	
Sample	**COLD**	**REG**	**COLD**	**REG**
1	■	□	■G12D	□
2	■	□	■G13D	□
3	■	□	■G12C	□
4	■	□	■G13R	□
5	■	■	■G12C	■G12C
6	■	■	■G12V	■G12V
7	■	■	■G12D	■G12D
8	■	■	■G12D	■G12D
9	■	■		■G13D
10	■	■		■G13D
11	■	■		■G13D
12	■	■		■G12D
13	■	■		■G12D
14	■	■		■G12R
15	■	■		■G12D
16	■	■		■G13D
17	■	■		■G12D
18	■	■		■G12D
19	■	■		
20	■	■		
21	■	■		
22	■	■		
23	■	■		
24	■	■/□		
25	■	■/□		
26	■	■/□		
27	■	■/□		
28	■	■/□		
29	■	■/□		
30	■	■/□		
31	■	■/□		
32	■	■/□		
33	■	■/□		
34	□	□	□	□
35	□	□	□	□
36	□	□	□	□
37	□	□	□	□
38	□	□		□
39	□	□		□
40	□	□		□
41	□	□		□
42	□	□		□
43	□	□		□
44	□	□		□
45	□	□		□
46	□	□		
47	□	□		
48	□	□		
49	□	□		
50	□	□		
51	□	□		
52	□	□		
53	□	□		
54	□	□		
55	□	□		
56	□	□		
57	□	□		
58	□	□		
59	□	□		
60	□	□		
61	□	□		

Black squares (■) indicate a *KRAS* mutation was detected; open squares (□) indicates no KRAS mutation detected. (■/□) indicates a low-positive *KRAS* result near the limit of detection. COLD= COLD-PCR; REG= Regular (conventional) PCR
