# Supplementary material for: COLD-PCR enhanced melting curve analysis improves diagnostic accuracy for KRAS mutations in colorectal carcinoma
Source: BMC Clin Pathol. 2010 Nov 26;10:6. doi: 10.1186/1472-6890-10-6 (PMC3001699; doi:10.1186/1472-6890-10-6)

Additional File 3: Regular- and COLD-PCR sequencing data for the 4 re-classified (discrepant) specimens.

This file contains sequencing results of regular- or COLD-PCR from the 4 discrepant (re-classified) specimens. Shown in each slide is a screenshot from Mutation Surveyor (Softgenetics) centered on *KRAS* exon 2 codons 12 and 13. Numbering across the top corresponds to genbank reference sequence NM_004985. The upper electropherogram trace corresponds to the reference wild-type *KRAS* sequence and the lower trace to the test sample. A difference plot at the bottom of each panel shows deviations between the sample and the reference sequence. Arrows point to the position of the mutation detected by COLD-PCR. Sample numbers correspond to Supplemental Table 1. To enhance mutation detection by DNA sequencing COLD-PCR was performed with a T_c of 79.4ºC for these samples as described in the methods section.

Contents:

Discrepant Sample #1: Slides 2-3
Discrepant Sample #2: Slides 4-5
Discrepant Sample #3: Slides 6-7
Discrepant Sample #4: Slides 8-9

Pritchard et al 2010

Discrepant Sample #1 COLD-PCR

KRAS Result = G12D (5571 G>A)

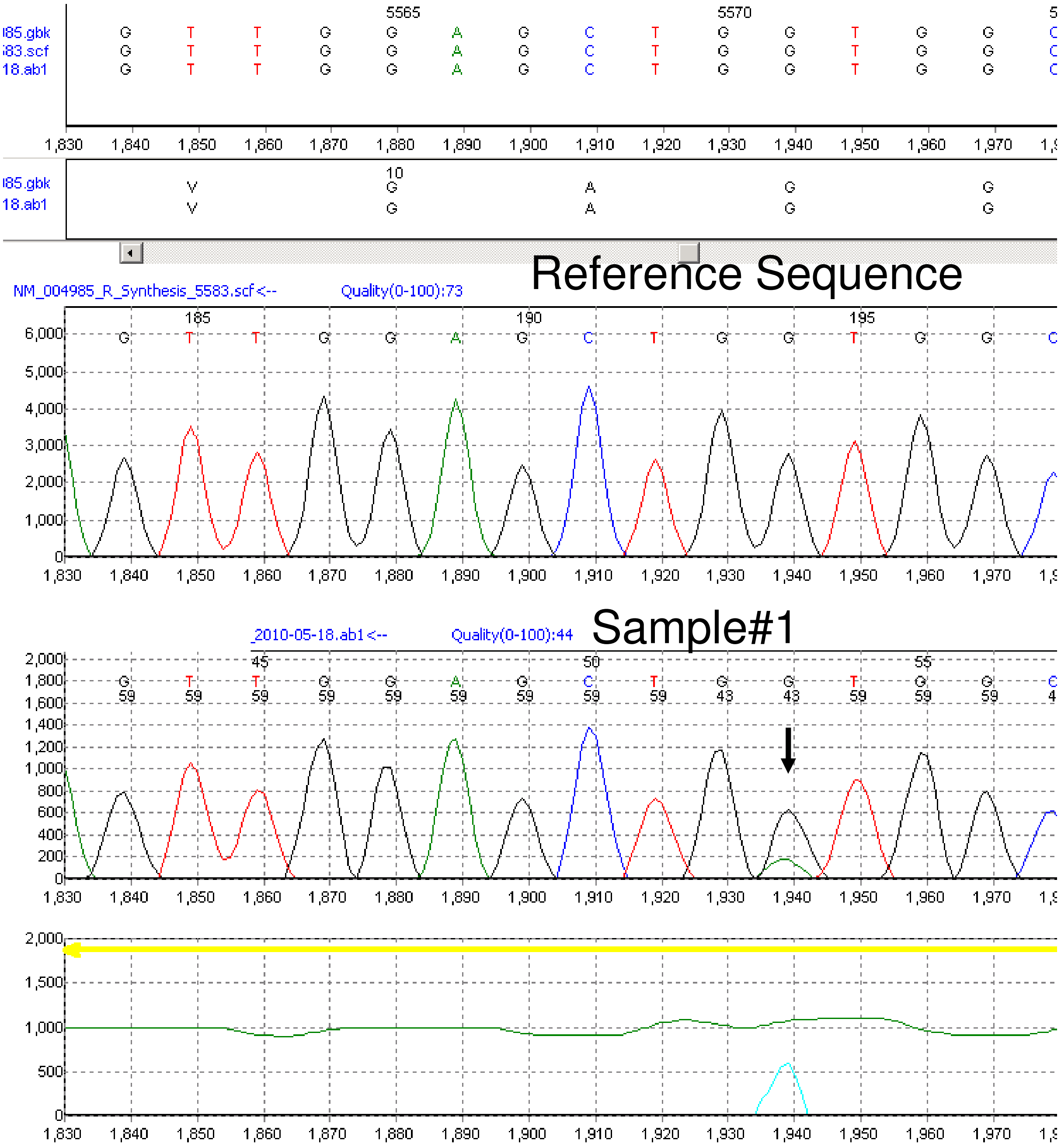

Discrepant Sample #1 Regular PCR

KRAS Result = Wild Type

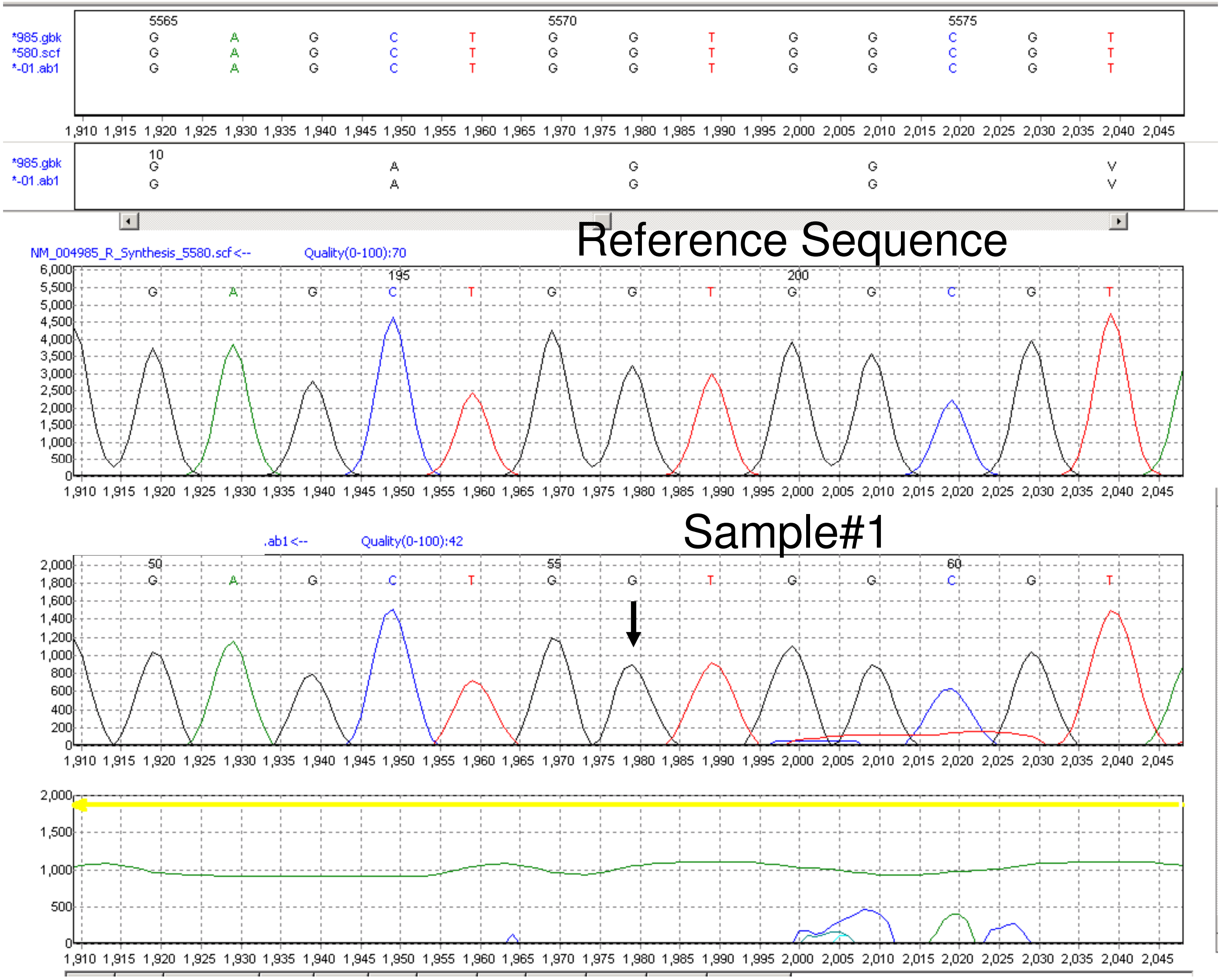

Discrepant Sample #2: COLD-PCR

KRAS Result = G13D (5574 G>A)

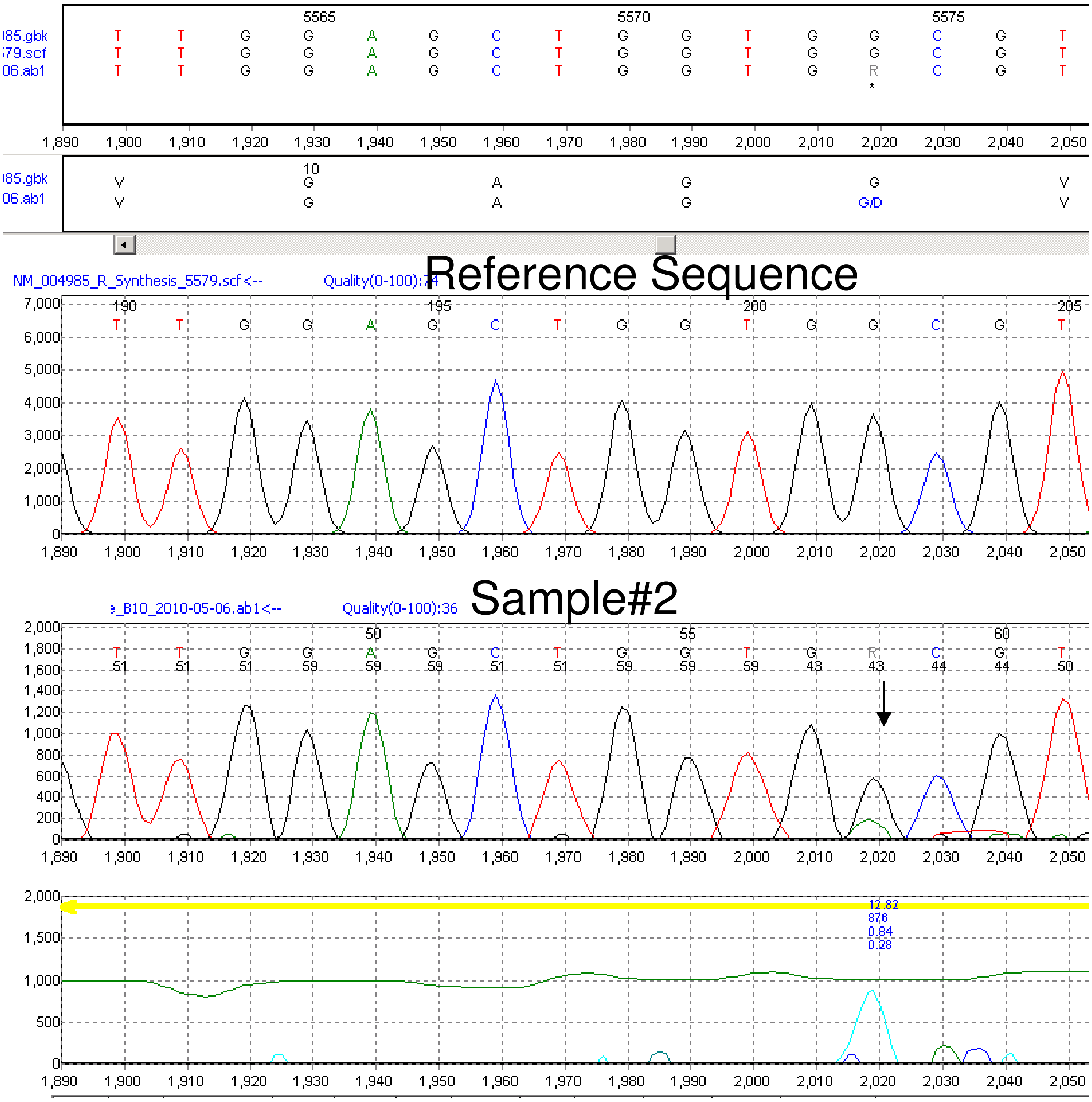

Discrepant Sample #2: Regular-PCR

KRAS Result = Wild Type

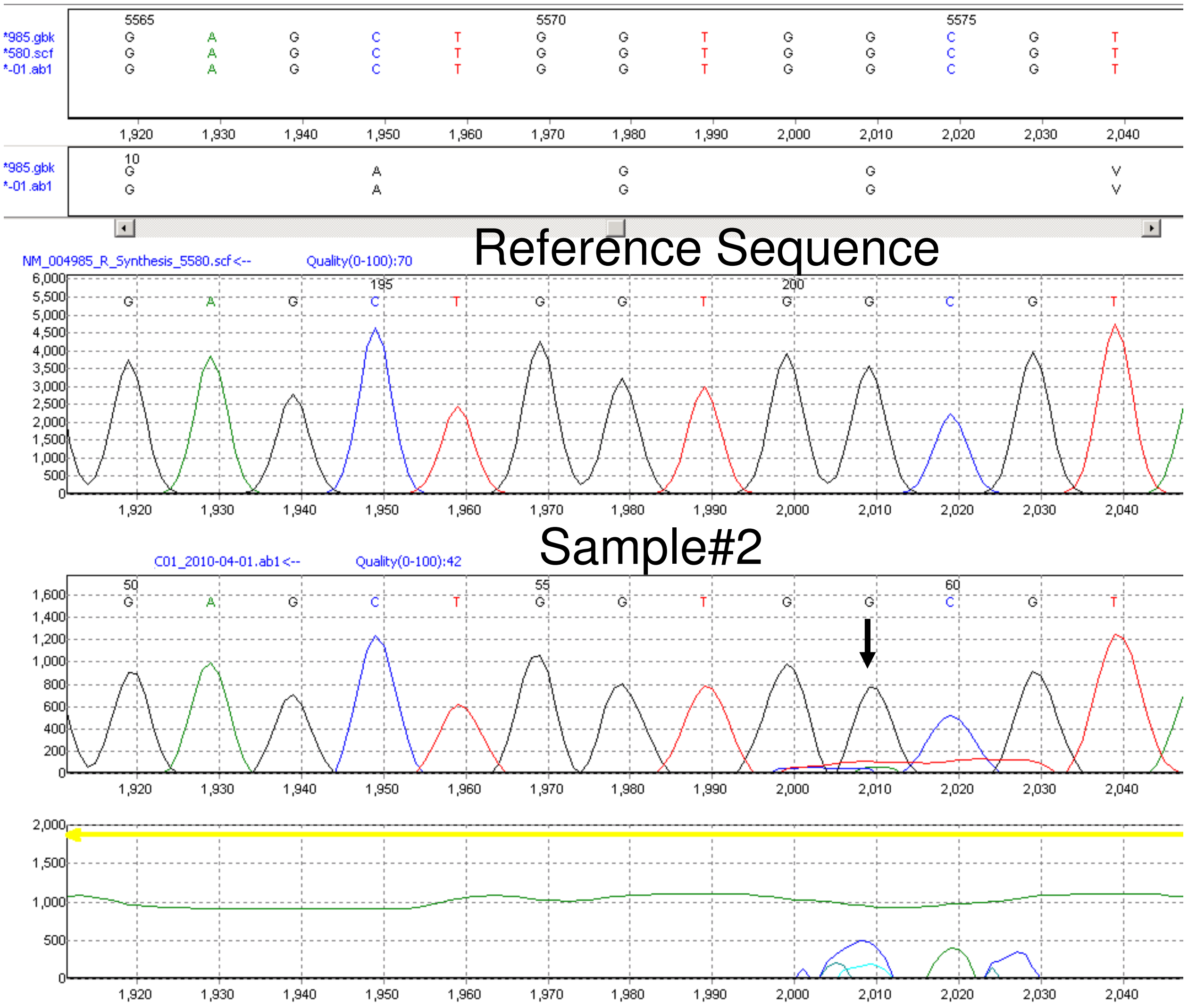

Discrepant Sample#3: COLD-PCR

KRAS Result = G12C (5570 G>GT)

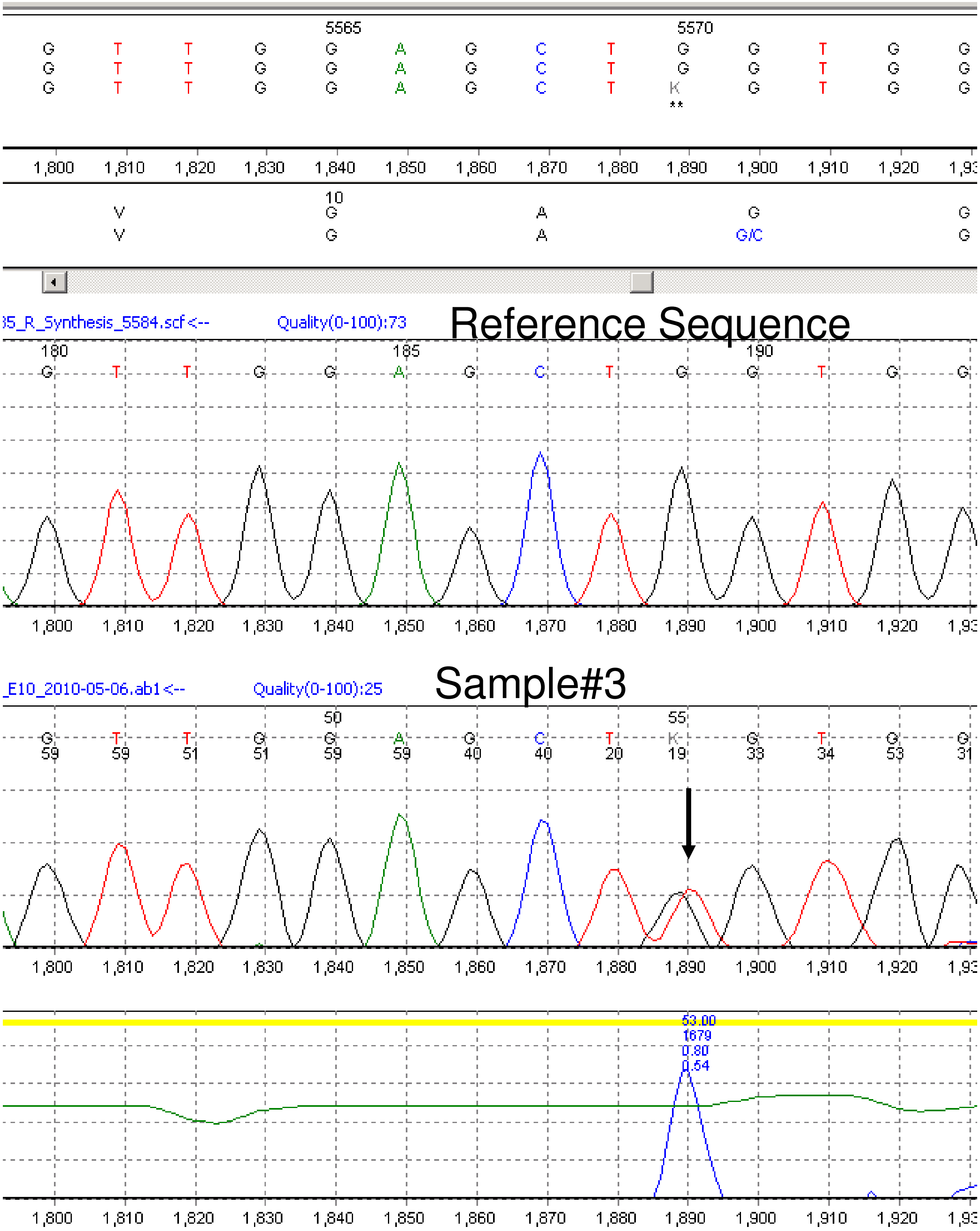

Discrepant Sample#3: Regular-PCR

KRAS Result = Wild Type

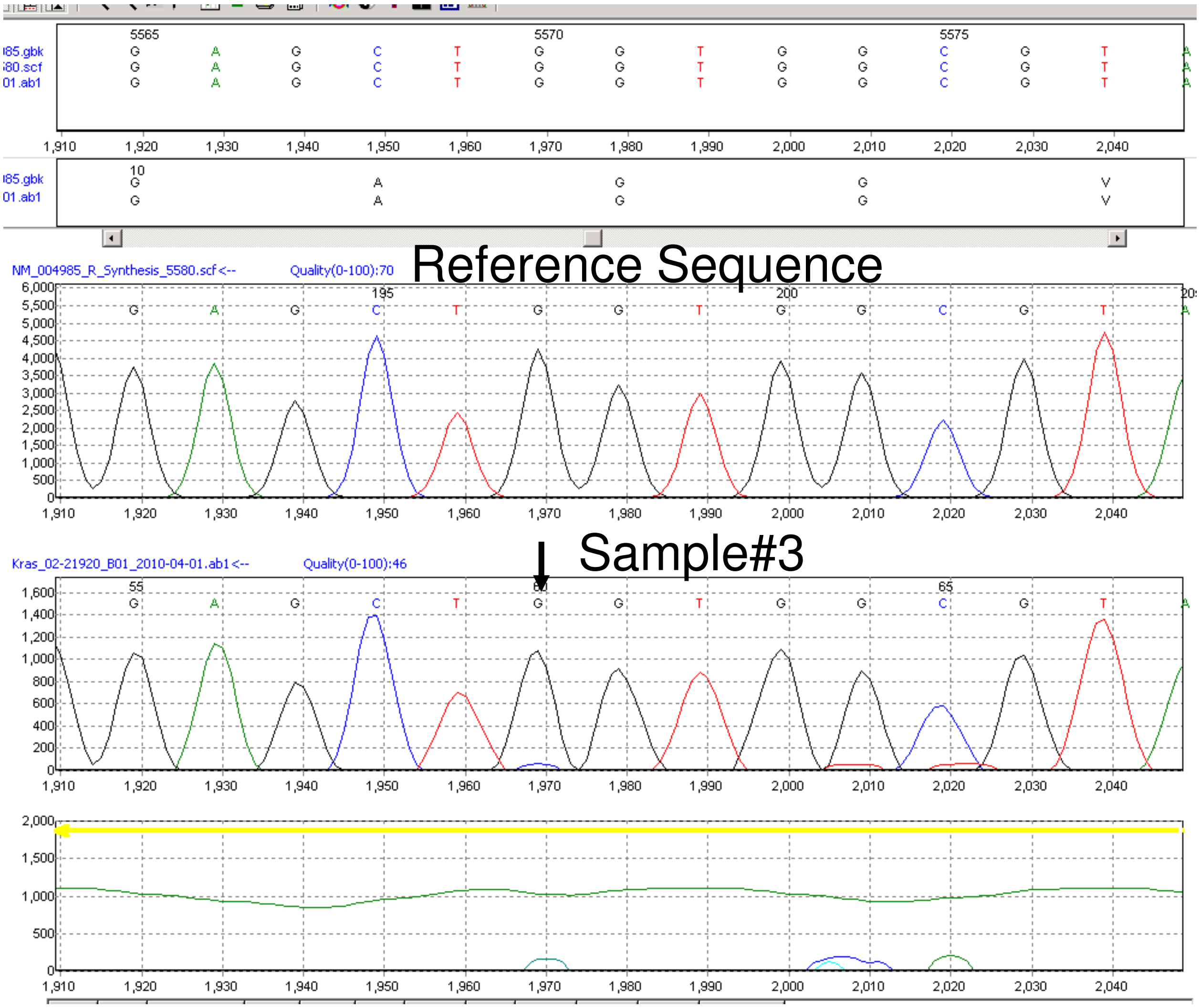

Pritchard et al 2010

Discrepant Sample #4: COLD-PCR

KRAS Result = G13R (5573 G>C)

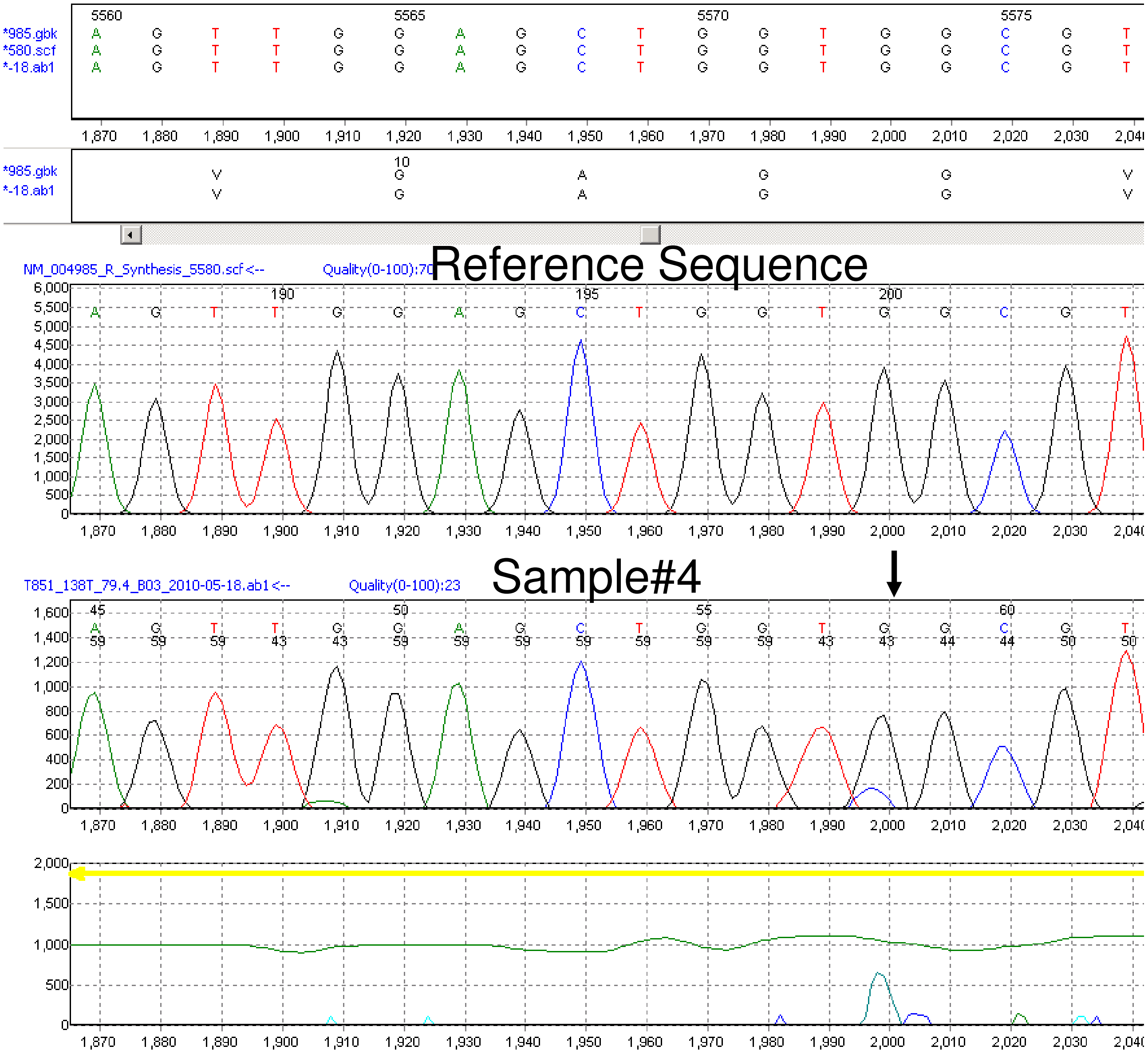

Discrepant Sample #4: Regular-PCR

KRAS Result = Wild Type

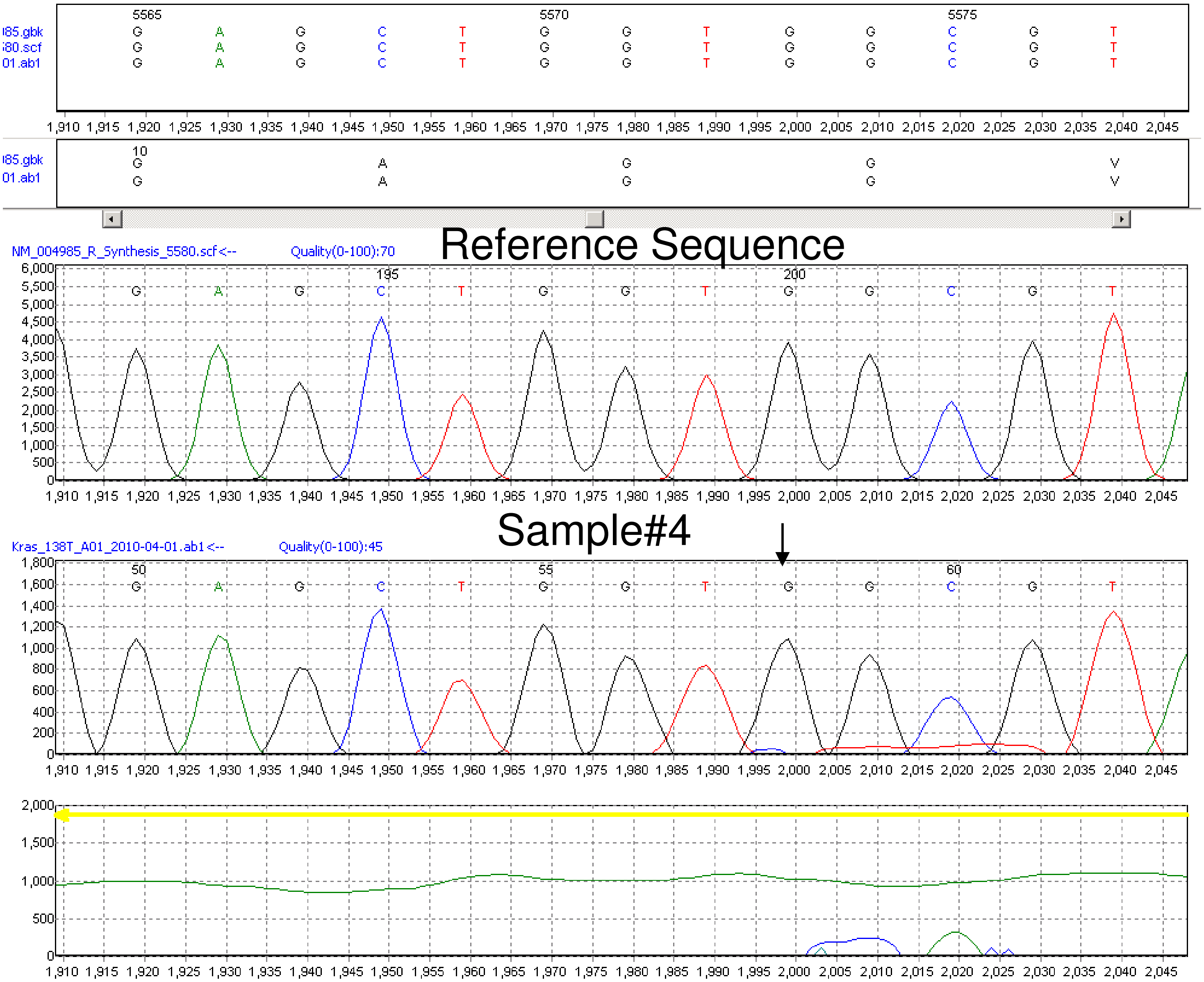

Supplement: Additional file 3 — Sequencing results on the re-classified specimens. Regular- and COLD-PCR sequencing data for the 4 re-classified (discrepant) specimens. [file 1472-6890-10-6-S3.PDF]
